# Perceptions of Large Wood in River Corridors: Visual Cues, Risk, and Management Expectations in Brazil

**DOI:** 10.1007/s00267-026-02489-y

**Published:** 2026-04-30

**Authors:** Bruno Henrique Abatti, Marina Refatti Fagundes, Gean Paulo Michel, Ronald E. Poeppl, Franciele M. Vanelli, Franciele Zanandrea, Karla Campagnolo, Leonardo Rodolfo Paul, Masato Kobiyama

**Affiliations:** 1 Hydraulic Research Institute, Federal University of Rio Grande do Sul, Grande do Sul, Brazil; 2 Institute of Hydrobiology and Aquatic Ecosystem Management, BOKU University, Vienna, Austria; 3 Department of Agricultural and Environmental Engineering, Fluminense Federal University, Rio de Janeiro, Brazil

## Abstract

Public perceptions of river corridors often diverge from scientific understanding, particularly regarding the ecological and geomorphological role of large wood (LW). This study examines how different societal and professional groups perceive large wood in Brazilian rivers using a structured photo-questionnaire. A total of 437 respondents evaluated images of river scenes with and without large wood across four perceptual dimensions (naturalness, aesthetics, hazard, and need for improvement). Large wood was frequently associated with flow obstruction, flood hazard, and the need for channel intervention, while relatively few respondents explicitly recognized its ecological functions. Comparisons between conditions showed statistically significant but modest effects, indicating that evaluations were strongly influenced by landscape context and image characteristics rather than by wood presence alone. Disciplinary background also affected responses, with participants from environmental and natural resource sciences showing greater acceptance of large wood. Overall, the results provide empirical evidence on how large wood is socially interpreted in river corridors and may inform discussions related to river management and restoration.

## Introduction

The scientific and technical communities have recently placed a strong emphasis on the importance of public engagement in achieving environmental management and sustainable development goals (UNDP, 2023; Fritz et al., 2019; Arheimer et al. 2024). This emphasis is particularly evident in the rise of citizen science, which enables society to actively participate in the generation of new knowledge and decision-making processes, thus expanding the reach of environmental awareness and promoting scientific dissemination (Buytaert et al. 2014; Starkey et al. 2017). Several authors, such as Albagli and Iwama (2022), McKinley et al. (2017), Vanelli and Kobiyama (2021), argue that citizen science can significantly strengthen public involvement in environmental issues and bridge the science–policy gap.

Public perception has been increasingly recognized as a key element in the integrated management of natural resources such as forests and rivers, helping to enhance the interface between science and policy (Fernández-Llamazares et al., 2016). However, transforming scientific findings into accessible and applicable information for public policy remains a significant challenge. Given the participatory nature of contemporary environmental governance, decisions related to river management increasingly extend beyond purely technical or scientific domains and are influenced by how river landscapes are perceived and interpreted by society. In this context, the scientific community cannot operate in isolation from broader public perceptions when addressing management strategies for fluvial systems (Reed, 2008). In this context, Environmental Education plays a central role in enabling society to understand environmental processes, interpret scientific evidence, and actively engage with environmental public policies, thereby fostering informed decision-making and long-term environmental stewardship (Ardoin et al., 2020). A fundamental disconnection often exists between scientific understanding and public perception, particularly in the context of complex environmental processes (Gregory et al., 2005; Aryal et al., 2021). Scientific research is normally conducted to produce structured, evidence-based knowledge that informs policy and decision-making (Holmes & Clark, 2008). However, the dynamic and interdependent nature of hydrological, biological, and social processes hampers the formulation of standardized guidance specifically for the management of Large wood in river corridors, as system responses vary markedly across spatial and temporal contexts (Wohl et al., 2019). This complexity constrains the development of practical management strategies for Large wood that are both effective and broadly applicable across different river settings.

In the realm of river corridor management, this complexity becomes particularly evident. From a conceptual perspective, the River Continuum Concept provides a foundational framework for understanding rivers as longitudinally connected systems, in which physical, chemical, and biological processes vary predictably from headwaters to downstream reaches (Vannote et al., 1980). As noted by e.g. Hooke (2023), the river corridor is a highly dynamic system influenced by processes across different spatial and temporal scales. Managers frequently request faster and more cost-effective assessment tools for basin-wide planning. Consequently, there is a growing demand for generalized approaches and guidelines applicable to rivers with similar hydrogeomorphic characteristics (Arthington et al., 2010). However, such generalized models risk overlooking the physical and management-specific particularities of individual river systems (Brierley et al., 2013; Poeppl et al., 2020).

Among the elements often overlooked or misunderstood in fluvial systems are natural disconnectivity features, such as Large wood (LW). In this study, we adopt the definition by Campagnolo et al. (2024), who describe LW as tree components, such as branches, trunks, or entire trees, that enter the river channel. Despite growing scientific recognition of the ecological and hydrogeomorphological significance of Large wood (LW) in river systems, conventional river management in many regions of the world, including Brazil, has historically emphasized the removal of such features from river channels (Campagnolo et al., 2020). Accumulating evidence now indicates that LW is not an anomalous or disruptive element, but a fundamental structural component of fluvial systems, whose abundance, size, mobility, and spatial configuration vary across climatic, geomorphological, and hydrological contexts, shaping river processes across multiple spatial and temporal scales (Wohl et al., 2019).

Through its interactions with flow and sediment, LW contributes to channel adjustment, habitat heterogeneity, and the development of hydrogeomorphological feedbacks, reflecting a broader shift in river science from viewing wood primarily as debris or hydraulic obstruction to recognizing it as a key driver of river dynamics and ecosystem functioning (Gurnell & Bertoldi, 2020; Swanson et al., 2020).

At the ecosystem level, LW supports a wide range of functions, including organic matter retention, nutrient and carbon cycling, regulation of water quality, and increased richness and diversity of aquatic and riparian biota (Verdonschot & Verdonschot, 2024). These functions are underpinned by process-level mechanisms such as the enhancement of hyporheic exchange, whereby localized hydraulic gradients and flow heterogeneity facilitate the transfer of heat, chemical solutes, and biota between surface and subsurface waters (Hester & Doyle, 2008). At the same time, recent syntheses emphasize that the ecological benefits associated with LW are strongly context-dependent and frequently involve management trade-offs, particularly in managed and urbanized river settings where concerns related to flood risk and channel conveyance remain prominent (Verdonschot & Verdonschot, 2024). In addition to its natural role, tree trunks and LW are increasingly used as active elements in river renaturalization and restoration projects, aiming to re-establish natural processes and enhance channel complexity, water quality, and aquatic biodiversity, including in tropical river systems (Pinto et al., 2017; Wohl et al., 2019).

The removal of such structures can have far-reaching consequences. As highlighted by Wohl and Beckman (2014), eliminating LW features may reduce organic matter storage and biological processing, with potential global impacts on carbon cycling. More broadly, the loss of structural elements that promote in-channel retention and localized disconnectivity has been shown to alter sediment and material routing in river networks (Poeppl et al., 2023; Galia et al., 2024; Abatti et al., 2024). From a hydraulic perspective, LW acts as a roughness element that reduces flow velocities, elevates the water level locally, improves floodplain connectivity, and increases flood wave travel time (Gippel, 1995). These hydraulic effects directly influence sediment transport, morphological development, pool–riffle formation, and overall stream stability (Piégay & Gurnell, 1997). Naturally, these features are more prevalent in fluvial systems with high transport capacity, such as steep or headwater reaches. When present in or near urbanized environments, however, LW may also require careful hydraulic evaluation, as wood recruitment, transport, and deposition during high-magnitude floods have been shown to increase the probability of bridge and culvert clogging and jam formation (Ruiz-Villanueva et al., 2014, 2017; Lyn et al., 2007), generate additional flow resistance and backwater effects that locally elevate flood stages and inundation extent (Comiti et al., 2016; Ruiz-Villanueva et al., 2017), and ultimately modify hazard patterns and risk estimates when wood dynamics are neglected in conventional flood assessments (Zischg et al., 2018; Ruiz-Villanueva et al., 2014).

While LW is widely recognized as a key structural component of river corridors under natural conditions, its ecological role and associated ecosystem services are strongly dependent on the processes governing its recruitment, transport, and redistribution along the river network. In river systems where longitudinal connectivity has been severely disrupted by large hydroelectric dams, the accumulation of dead tree trunks may no longer reflect a natural wood regime, but rather the legacy of artificial flooding and interrupted downstream transport. In tropical rivers affected by major reservoirs, such as those associated with the Balbina and Tucuruí dams in Brazil, extensive inundation of forested areas has resulted in large volumes of standing and fallen dead wood persisting along reservoirs and channel margins (Fearnside, 1999). Unlike naturally recruited LW in free-flowing rivers, this excessive and spatially homogeneous accumulation is not dynamically reworked by fluvial processes, which can alter ecosystem functioning. Previous studies indicate that such conditions may negatively affect regulating ecosystem services, including biogeochemical cycling, water quality, and carbon dynamics, particularly through enhanced greenhouse gas emissions associated with wood decomposition in reservoirs (Barros et al., 2011; Fearnside & Pueyo, 2012). These examples illustrate that the presence of wood in rivers cannot be evaluated in isolation from connectivity conditions and disturbance history, reinforcing the need to distinguish between natural disconnectivity features and artificial accumulations when interpreting both ecological outcomes and societal perceptions.

Given this scientific understanding, the gap between knowledge and practice becomes concerning, especially in contexts where environmental governance lacks regulatory frameworks addressing these elements, as is, for instance, currently/still the case in Brazil. The increasing awareness of the complexity of human–water systems reinforce the need for innovative management approaches that incorporate social perception and foster participatory, integrated decision-making (Pahl-Wostl, 2007; Montanari et al., 2013). Brazil’s National Water Resources Policy was developed on the principles of decentralization and the active participation of institutional actors, service users, and civil society, and incorporating public perception into water resources management is consistent with these principles. Assessing perception offers valuable evidence that supports planning, implementation, and adaptive management of natural resources (Bennett, 2016). Themes like river restoration and riparian forest management remain underexplored in the legal and practical context in Brazil. Consequently, this research seeks to contribute to the broader discourse on water resource governance by providing empirical evidence on how local perceptions influence, and can support, the development of effective, community-driven policies for river re-naturalization and riparian forest management.

Inspired by the pioneering photo-questionnaire approach of Piégay et al. (2005), a growing body of research has examined how LW is socially perceived across different cultural, educational, and hydrological contexts. Collectively, these studies indicate that evaluations of LW tend to cluster around visual aesthetics, perceived naturalness, safety concerns, and the perceived need for channel “improvement”, while varying according to respondents’ background, familiarity with river processes, and previous flood experience (Chin et al., 2008; Le Lay et al., 2008; Wyżga et al., 2009; Wohl, 2015; Ruiz-Villanueva et al., 2018; Gapinski et al., 2021; Dalu et al., 2022). More recent syntheses further emphasize that this perception gap remains a key barrier to the implementation of wood-based restoration and risk-informed river management strategies (Wohl, 2015, 2016; Ockelford et al., 2024). In this context, we investigate how a diverse sample of professional and academic stakeholders in Brazil, including scientists, policymakers, and respondents with technical backgrounds, perceive disconnectivity features within river corridors, particularly those associated with LW.

## Methodology

### Survey Design and Data Collection Procedures

This study adopted a perception-based approach centred on the visual evaluation of riverine landscapes in Brazil. Participants were required to assess photographs of rivers with and without the presence of disconnectivity features, particularly LW elements, following the methodology proposed by Piégay et al. (2005) and Chin et al. (2008). Prior to the visual assessment, participants were provided with brief and generic definitions of each perception criterion on the first page of the questionnaire, aimed solely at ensuring a shared semantic understanding of the terms. These definitions included: *Aesthetic* (harmony of forms and/or colours; beauty), *Naturalness* (quality or condition of what is natural), *Hazard* (a situation posing a threat to the integrity or safety of people, animals, or objects), and *Need for Improvement* (the need for change toward a better state or condition). No technical or management-oriented explanations were provided in order to avoid influencing individual judgements. As Piégay et al. (2005) noted, the use of standardized photographs is a scientifically consolidated method for assessing perceptual judgements in environmental contexts, especially when investigating public attitudes towards river management. For this, only photographs of Brazilian rivers were used, which could reinforce the cultural and environmental familiarity of the Brazilian respondents with the fluvial corridor. This approach was adopted to minimize interpretation bias and ensure that respondents could recognize common features, such as vegetation, channel width, and river morphology, inherent to their geographic and ecological background.

The survey was conducted and structured into two phases. First, participants provided information on their own socio-professional background, including education level, field of expertise, current occupation, and residence place. Both levels of education and occupation were defined as single-choice variables, ensuring that each respondent was assigned to only one category per variable. This information enabled the classification of respondents into different analytical groups. Then, participants evaluated 10 photographs through a structured online photo-questionnaire. For each image, the four perception dimensions (Aesthetic, Naturalness, Hazard, and Need for Improvement) were assessed independently using direct questions displayed below the photograph. Responses were provided using a three-level, star-based Likert-type scale representing low, moderate, and high intensity. These visual categories were subsequently converted into ordinal scores (1–3) for statistical analysis. Given the online format and the repetitive nature of the task, requiring multiple rapid evaluations across several images, the reduced number of response options was adopted to facilitate intuitive judgements, reduce cognitive burden, and minimize respondent fatigue and survey abandonment. Broader methodological implications of this choice are discussed in the Discussion section.

For items related to Hazard and Need for Improvement, whenever participants selected options indicating concern (score 2 or 3), they were required to justify their response via an open-ended field and to optionally suggest potential management actions or modifications to the related site/photograph. At the end of the visual evaluation, participants answered a final open question: “What do you think about the presence of wood elements (trees, trunks, and branches) inside river channels?” This was followed by a prompt for a written justification regarding whether they considered LW to be beneficial or detrimental, and why.

The questionnaire was administered exclusively through a digital platform to ensure standardized layout, content, and instructions while enabling broad participation across Brazil. Recruitment followed a voluntary, non-probabilistic convenience sampling approach. The survey link was distributed through academic mailing lists, institutional networks, and professional contacts, particularly among governmental agencies, universities, and private companies working in environmental management and engineering. Participation was open to adults residing in Brazil, and responses from individuals outside these sectors were also accepted to capture a broader range of perspectives. The survey remained available throughout 2024. To preserve methodological rigour, the questionnaire instructions and introductory section were intentionally framed in neutral terms prior to the visual assessment, avoiding any explicit mention of river restoration, wood in rivers, or related management concepts that could bias participants’ initial judgements. Participants were informed only that the survey aimed to assess perceptions of river environments based on photographs. This neutral framing was designed to ensure that first impressions and perceptual responses remained spontaneous, shaped primarily by respondents’ previous experiences and intuitive interpretations. Questions explicitly referring to wood were presented only after the completion of the image-based evaluations.

### Selection of the Photographs

**Fig. 1:**
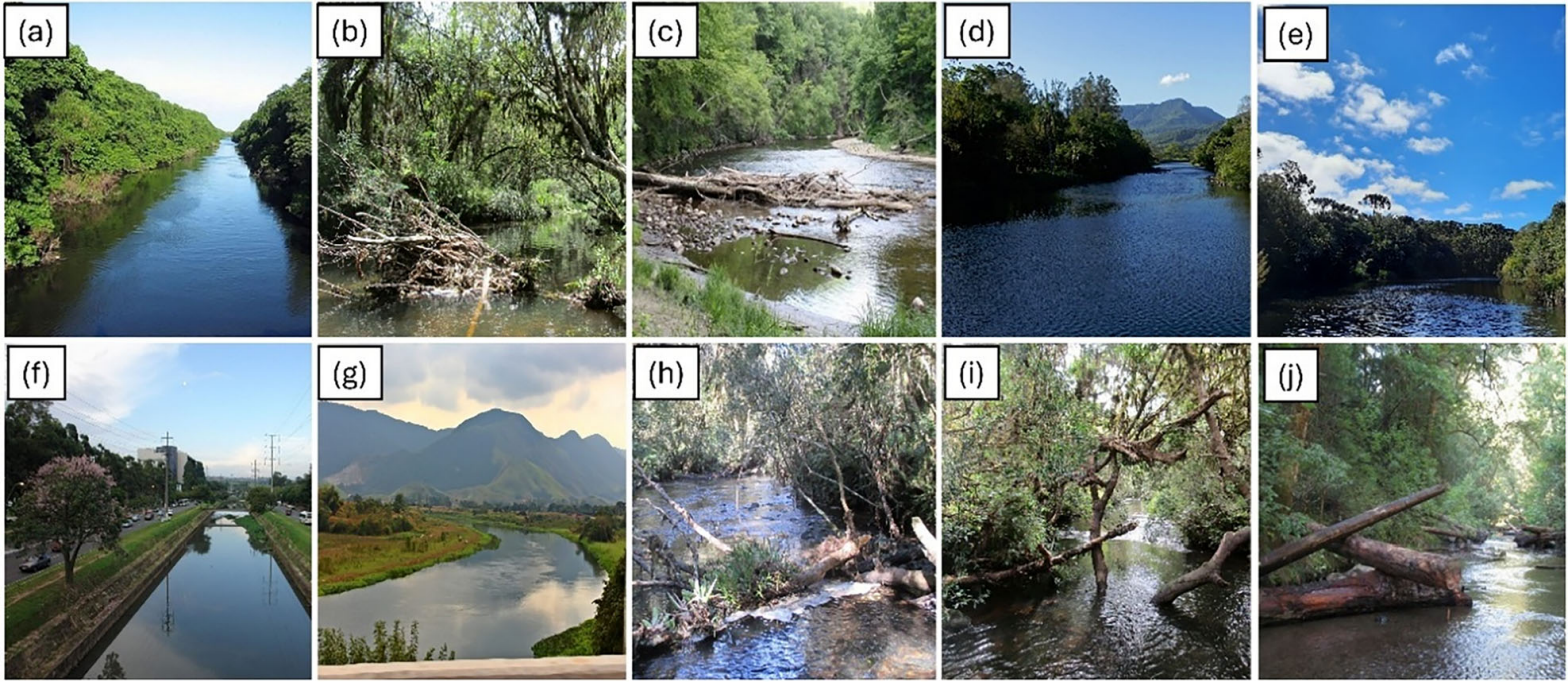
Photographs used in the visual perception survey. Panels (**a**-**j**) depict river corridor conditions, with (**a**, **d**, **e**, **f**, **g**) showing channels without large wood (LW) and (**b**, **c**, **h**, **i**, **j**) showing channels with LW.

The images used in this study were primarily selected from photographic records of Brazilian rivers, with an emphasis on the southern region of the country, where the Mixed Ombrophilous Forest (Araucaria Forest) predominates. Image A represents the Cocó River, located in Fortaleza, Ceará, within the Caatinga biome, characterized by a semi-arid landscape and a strong socio-cultural relationship with water scarcity. Image G depicts the Guandu River, in Rio de Janeiro, within the Atlantic Forest biome, in a highly urbanized and managed river context. The remaining eight images represent rivers from the Serra Geral region, in southern Brazil (Rio Grande do Sul), also within the Atlantic Forest biome, predominantly in the Mixed Ombrophilous Forest domain, characterized by mountainous landscapes, dense riparian vegetation, and relatively preserved fluvial corridors. This geographic focus allowed the representation of channel settings characteristic of this vegetation type, including its specific hydromorphological and riparian features. In addition to the predominant focus on southern Brazilian rivers, a limited number of images from other regions were intentionally included, selected not only for their visual quality but also for their clear depiction of contrasting riverbank conditions, channel morphology, and levels of urban influence (Fig. 1). This decision aligns with recommendations by Piégay et al. (2005) who emphasized the importance of avoiding unfamiliar or foreign landscapes that may bias perception studies. Images were carefully composed to focus primarily on the river channel and its immediate surroundings (river corridor). Elements such as bridges, signs, or infrastructure were deliberately excluded to prevent interpretive interference. The selection strategy aimed to facilitate the assessment of natural features, such as LW within rivers, channel form, and riparian vegetation, while avoiding contextual elements that could suggest previous human management or potentially influence the perception of risk. Although the study did not aim to assess perceptions of explicitly urban river environments, the image set was designed to provide a neutral and diverse representation of Brazilian river corridors rather than to contrast urban versus natural settings. It is nevertheless acknowledged that respondents may project risk-based interpretations shaped by prior experiences in urban or engineered contexts, even when evaluating images depicting relatively natural or less modified river reaches.

### Statistical Analysis

All analyses were conducted at the respondent level to ensure independence among observations. Because each participant evaluated multiple photographs, perceptual scores were aggregated by calculating the mean value of images containing LW and those without LW for each perceptual dimension. This procedure resulted in one representative score per participant and condition and follows approaches commonly adopted in photo-based perception studies (e.g., Ruiz-Villanueva et al., 2018).

Descriptive statistics (mean, median, standard deviation, and frequency distributions) were computed to summarize overall perceptual tendencies. For descriptive purposes, summaries were also reported at the evaluation level (all responses pooled across images) to characterize general trends across stimuli.

As responses were recorded on a 3-point Likert-type scale and treated as ordinal data, non-parametric statistical methods were applied. Differences between the two image conditions (LW vs. non-LW), evaluated within the same respondents, were assessed using the Wilcoxon signed-rank test. Differences among categorical socio-professional groups (education level, area of expertise, and employment sector) were evaluated using the Kruskal–Wallis test. Associations among perceptual dimensions (Aesthetics, Naturalness, Hazard, and Need for Improvement) were assessed using Spearman’s rank correlation coefficient (ρ).

## Results

### General perceptions regarding the presence of large wood in rivers

A total of 437 complete and valid responses were collected and included in the analysis. The final dataset comprised individuals from diverse regions and professional backgrounds, enabling structured comparisons between societal groups with varying degrees of technical knowledge and engagement in environmental issues. Respondents were geographically distributed across 19 of the 27 Brazilian states. The sample was markedly concentrated in the southern region, particularly in the states of Santa Catarina (SC, 44.39%) and Rio Grande do Sul (RS, 23.80%), which together accounted for 68.19% of all responses. Additional notable contributions were from Mato Grosso do Sul (MS, 11.21%) and Minas Gerais (MG, 8.92%). Other states, including Rio de Janeiro (RJ, 3.20%), Pará (PA, 1.37%), Alagoas (AL, 1.14%), Paraná (PR, 1.14%), and São Paulo (SP, 1.14%), exhibited lower representation. The remaining states each comprised less than 1% of the sample. This distribution evidence a regional bias toward the southern portion of the country, which should be considered when interpreting the results, particularly regarding potential regional differences in environmental perception and familiarity with fluvial systems containing LW. This pattern is primarily associated with higher response uptake in regions where the research group has a stronger institutional presence, despite nationwide dissemination of the questionnaire. Table 1 presents a general summary of the characteristics of the respondents who completed the questionnaire. Given the predominantly virtual dissemination of the survey, the respondent profile reflects a higher level of engagement with academic and professional environmental contexts.

**Table 1:** Overview of respondents’ characteristics (*n* *=* 437)

Variable	Frequency (*n*)	Percentage (%)
**A: Level of education**		
Completed secondary education	10	2.29%
Undergraduate degree in progress	47	10.76%
Completed undergraduate degree	161	36.84%
Master degree	125	28.60%
Doctor's degree (PhD)	94	21.51%
**B: Area of expertise**		
Environmental and natural resource sciences	164	37.53%
Engineering and technology	118	27.00%
Health and multidisciplinary sciences	51	11.67%
Social sciences, humanities, and education	104	23.80%
**C: Employment sector**		
Researcher	107	24.49%
Public sector employee	142	32.49%
Private sector employee	125	28.60%
Student	59	13.50%
Retired	4	0.92%

The evaluation of LW within riverine environments revealed a predominantly unfavourable perception among respondents. Based on the analysis of responses (*n* = 437), 70% of the participants classified the presence of LW as negative, while only 17% considered it positive, and 13% declared no clear opinion. To better characterize the rationale behind these assessments, participants were asked to indicate the primary motivations for their classification. Among those with negative evaluations, the most frequently reported concern was the increased flood risk associated with the obstruction of flow (50%), followed by the perception of disconnectivity of the river system (25%) and the idea of LW as a pollutant of water resources (16%). A residual portion (9%) selected the “Other” category, which included open-ended mentions of visual discomfort or a sense of neglect.

**Fig. 2:**
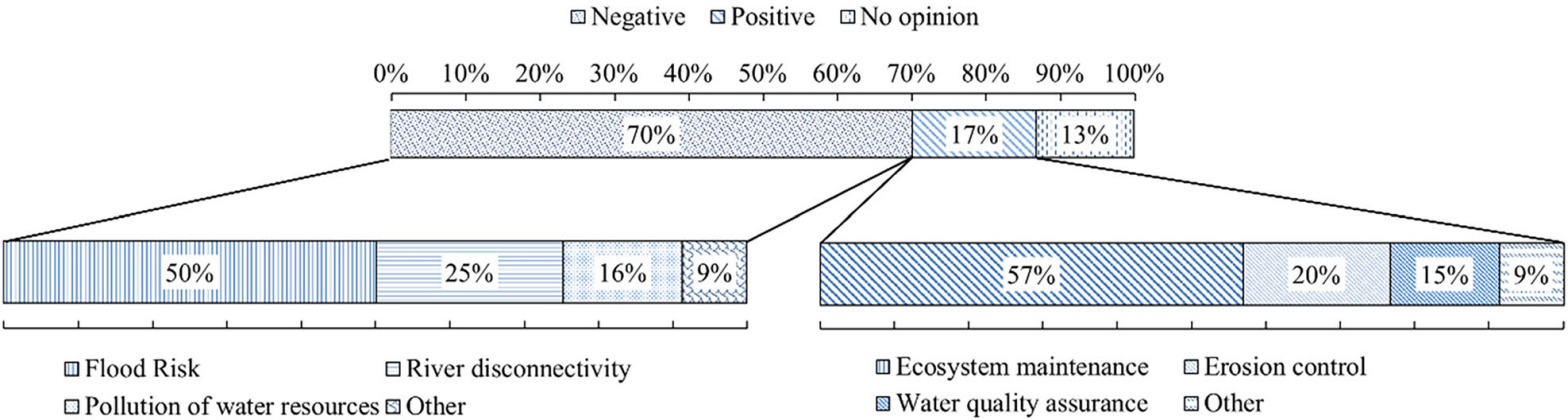
General perceptions and justification categories regarding the presence of LW in rivers: Overall classification of LW presence as negative, positive, or neutral by participants (*n* = 437)

On the other hand, respondents who evaluated the presence of LW positively justified their responses primarily by recognizing its ecosystem functions: 57% highlighted its importance for habitat maintenance and ecological integrity, 20% referred to its role in erosion control, and 15% mentioned benefits for water quality enhancement. A minority (9%) provided alternative justifications, most notably referencing the role of LW as a refuge or shelter for aquatic and riparian fauna. Fig. 2 represents the general opinions and the reasons given by participants for considering LW as either a positive or a negative element within river systems.

These results demonstrate that, while a segment of the respondents acknowledges the ecohydrological relevance of LW, the majority still perceive it as a risk element or a symptom of river degradation. This implies a substantial gap between respondent perception and scientific consensus on the functional role of LW elements in fluvial systems. However, such risk-based associations appear to be broadly generalized across river types, including low-order streams and less urbanized catchments, where the actual risk is comparatively limited. Because LW is systematically removed from highly urbanized river sections as part of routine maintenance and flood risk management, its presence is predominantly associated with less urbanized and more natural riverine environments. Consequently, image-based perception studies inherently reflect the typical environmental contexts in which LW occurs, rather than its presence in isolation. Importantly, this contextual association is central to the scope of the present study, which seeks to evaluate whether LW is socially accepted in riverine settings where its occurrence is ecologically expected and functionally beneficial. While concerns related to LW in urban contexts may be contextually justified, the persistence of unfavourable perceptions in predominantly natural environments highlights a key gap between ecological function and social interpretation. Furthermore, participants rarely demonstrated critical trade-off reasoning when justifying their responses. For example, the relative assessment of ecological benefits versus potential hazards was seldom addressed, which indicates a predominantly unidimensional interpretation of LW presence.

### Perception Patterns Across Areas of Expertise, Employment Sectors and Educational Level

Perceptual responses were compared across three grouping variables: (i) employment sector; (ii) area of expertise; (iii) and education level. Differences among groups were assessed using Kruskal–Wallis tests applied to respondent-level mean scores aggregated across images with and without LW (Table 2). Statistically significant differences were observed across all three variables. The strongest and most consistent effects were associated with area of expertise (significant differences across Aesthetics, Naturalness, Hazard, and Need for Improvement; *p* ≤ 0.01), whereas employment sector and education level showed significant differences mainly for specific dimensions, particularly Aesthetics and perceived Need for Improvement (*p* < 0.05).

**Table 2:** Mean ± SD perceptual scores by education level, area of expertise and employment sector. Δ represents the difference between LW and non-LW image evaluations (LW – no LW)

Variable	Naturalness (mean ± SD, Δ)	Aesthetics (mean ± SD, Δ)	Hazard (mean ± SD, Δ)	Improvement (mean ± SD, Δ)
**A: Level of Education**				
Completed Undergraduate Degree	2.50 ± 0.64 (+0.18)	2.22 ± 0.68 (−0.21)	1.80 ± 0.71 (+0.09)	1.76 ± 0.73 (+0.17)
Master Degree	2.53 ± 0.62 (+0.20)	2.25 ± 0.66 (−0.19)	1.76 ± 0.69 (+0.07)	1.73 ± 0.72 (+0.15)
DoctorDegree (PhD)	2.58 ± 0.60 (+0.23)	2.30 ± 0.63 (−0.16)	1.72 ± 0.67 (+0.05)	1.68 ± 0.70 (+0.13)
Undergraduate Degree in Progress	2.46 ± 0.66 (+0.16)	2.21 ± 0.69 (−0.20)	1.81 ± 0.72 (+0.10)	1.78 ± 0.74 (+0.19)
Completed Secondary Education	2.30 ± 0.70 (+0.12)	2.15 ± 0.73 (−0.18)	1.85 ± 0.76 (+0.14)	1.88 ± 0.79 (+0.23)
**B: Area of expertise**				
Environmental and Natural Resource Sciences	2.60 ± 0.59 (+0.23)	2.34 ± 0.62 (−0.14)	1.65 ± 0.66 (+0.02)	1.61 ± 0.69 (+0.08)
Engineering and Technology	2.45 ± 0.66 (+0.15)	2.18 ± 0.69 (−0.25)	1.86 ± 0.73 (+0.12)	1.84 ± 0.74 (+0.22)
Social Sciences, Humanities, and Education	2.47 ± 0.65 (+0.17)	2.20 ± 0.67 (−0.21)	1.83 ± 0.71 (+0.10)	1.79 ± 0.73 (+0.18)
Health and Multidisciplinary Sciences	2.49 ± 0.68 (+0.18)	2.23 ± 0.70 (−0.19)	1.78 ± 0.74 (+0.08)	1.76 ± 0.75 (+0.17)
**C: Employment Sector**				
Researcher	2.55 ± 0.62 (+0.21)	2.29 ± 0.65 (−0.18)	1.72 ± 0.69 (+0.05)	1.70 ± 0.71 (+0.15)
Public Sector Employee	2.48 ± 0.66 (+0.17)	2.23 ± 0.67 (−0.20)	1.80 ± 0.72 (+0.09)	1.77 ± 0.73 (+0.17)
Private Sector Employee	2.50 ± 0.63 (+0.18)	2.21 ± 0.68 (−0.22)	1.82 ± 0.70 (+0.10)	1.78 ± 0.74 (+0.19)
Student	2.47 ± 0.70 (+0.16)	2.24 ± 0.71 (−0.19)	1.75 ± 0.73 (+0.07)	1.74 ± 0.76 (+0.16)
Retired	2.40 ± 0.75 (+0.10)	2.20 ± 0.72 (−0.20)	1.90 ± 0.78 (+0.15)	1.95 ± 0.80 (+0.25)

Employment sectors showed significant differences primarily for Aesthetics and Need for Improvement (*p* < 0.05). However, the three largest groups, public sector employees, private sector employees, and researchers, which together comprised most of the sample, exhibited broadly similar response patterns. Across these groups, LW photos tended to receive slightly lower Aesthetic ratings compared to those without LW photos. Naturalness ratings remained neutral to slightly positive across sectors, and Hazard perceptions varied only minimally. Higher Need for Improvement scores were observed in smaller subgroups, particularly retirees, although these values should be interpreted cautiously given the limited sample size. Overall, the employment sector explained only moderate variation in LW perception. Clearer differences emerged across disciplinary backgrounds. Respondents from Environmental and Natural Resource Sciences, the largest expertise group, consistently attributed higher Naturalness and Aesthetic scores to LW photos and reported lower hazard and intervention demands. Engineering and Technology participants exhibited intermediate values. In contrast, respondents from Health and Multidisciplinary Sciences and from Social Sciences, Humanities, and Education tended to assign lower Aesthetic and Naturalness ratings and expressed higher perceived Hazard and stronger Need for Improvement. Differences across expertise groups were statistically significant for all perception dimensions (*p* ≤ 0.01). Education level was also associated with LW perception, although effects were less pronounced than those observed for expertise. Significant differences were detected primarily for perceived Need for Improvement (*p* < 0.05). Respondents with lower formal education levels tended to report higher intervention demands, whereas participants with postgraduate degrees (Master’s and PhD) generally showed lower improvement scores and slightly higher naturalness evaluations.

### Descriptive Analysis of Image Perception

**Fig. 3:**
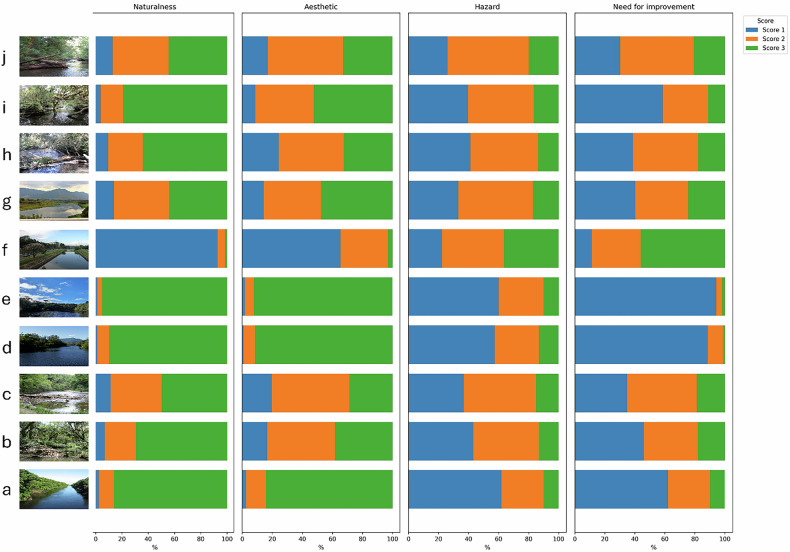
Percentages of respondents across the rating scores (1–3) for each perceptual attribute, considering all evaluated images. In this scale, 1 indicates a low level of the attribute, 2 a moderate level, and 3 a high level

The perceptual assessment of ten images of river, based on four key visual attributes (Naturalness, Aesthetic, Hazard, and Need for Improvement) revealed systematic differences between images that contained LW and those that did not. Fig. 3 presents the percentages of respondents across the rating scores for each perceptual attribute, for all evaluated images.

Overall, images without LW (a, d, e, f, and g) received higher scores for Naturalness and Aesthetics, although with notable variability among them. Images e and d were rated as highly natural by 95.2% and 89.5% of participants, and as highly aesthetic by 92.2% and 91.5%, respectively. These images also showed very low perceived hazard and minimal intervention need, with less than 2% of respondents assigning the highest scores in those attributes.

In contrast, Image g, despite not containing wood, received moderate perceptual evaluations: only 44.2% of participants rated it as highly natural and 47.6% as highly aesthetic. Furthermore, 17.2% considered it highly hazardous, and 24.5% indicated a strong need for intervention. The image depicts a river section with partial riparian degradation and small signs of anthropogenic influence, possibly related to nearby agricultural activity. Although the landscape may still appear relatively natural, the lack of riparian vegetation continuity and subtle land-use marks seems to have shaped more cautious evaluations by the public.

A stronger divergence was observed in Image f, also without LW, which received very low ratings for Naturalness (1.4%) and Aesthetics (3.0%). Most participants (92.9%) considered it not natural, and 65.4% assigned the lowest aesthetic value. Moreover, 36.6% rated it as highly dangerous, and 56.1% perceived a high need for improvement. This image demonstrates a rectified channel in a densely urbanized area, with visible infrastructure and modified banks, which evidently reinforced perceptions of degradation, risk, and loss of ecological quality.

Conversely, images containing LW (b, c, h, i, and j) exhibited greater variability and overall lower evaluations in Naturalness and Aesthetics. For instance, only 49.7% of participants rated Image c as highly natural, and just 28.6% perceived it as highly aesthetic. This image shows a cluttered and structurally complex fluvial setting, in which the arrangement and quantity of LW may have amplified impressions of disorder or instability. Similarly, Image j received high ratings for naturalness and aesthetic value from only 44.4% and 32.7% of participants, respectively. These values are considerably lower than those observed in most wood-free images. Moreover, although 19.9% rated the image as highly hazardous and 20.8% indicated a high need for improvement, these proportions are far above those for images without wood, where such ratings were typically below 2%.

Nevertheless, not all images with LW were perceived negatively. Image i, for example, was rated as highly natural by 79.2% of participants and aesthetic by 52.4%, suggesting that contextual landscape features, such as riparian cover and visual balance, may mitigate the negative associations typically attributed to LW. Similarly, Image h reached 64.1% for naturalness and 32.5% for aesthetics.

### Comparative Evaluation of Images With and Without LW

Perceptual scores were first averaged at the respondent level for images containing LW and for those without LW. Differences between conditions were assessed using the Wilcoxon signed-rank test.

Naturalness ratings were slightly higher for images containing LW (mean = 2.53, median = 3, SD = 0.65) compared to those without LW (mean = 2.41, median = 3, SD = 0.83), and this difference was statistically significant (*p* < 0.001). In contrast, Aesthetic evaluations were significantly lower for LW images (mean = 2.20, median = 2, SD = 0.71) than for images without LW (mean = 2.47, median = 3, SD = 0.77; *p* < 0.001). Hazard perception showed a small but statistically significant increase in LW scenes (mean = 1.79, SD = 0.70) relative to wood-free scenes (mean = 1.71, SD = 0.75; *p* = 0.002). The largest difference was observed for Need for Improvement, which was significantly higher for LW images (mean = 1.76, median = 2, SD = 0.73) than for images without LW (mean = 1.53, median = 1, SD = 0.78; *p* < 0.001). Standard deviations were similar across conditions (SD 0.65–0.83), indicating comparable dispersion of responses. Although statistically significant differences were detected between LW and non-LW images, individual ratings remained variable across respondents.

**Fig. 4:**
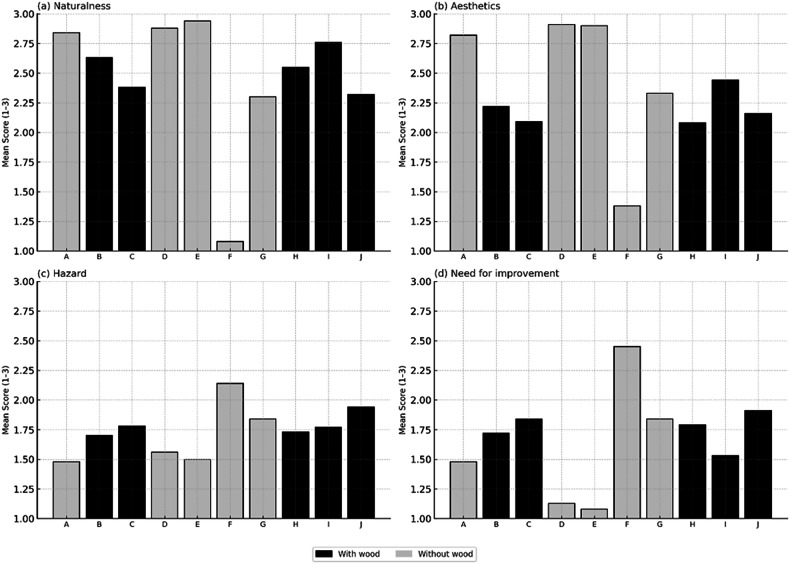
Perceptual responses to images with and without large wood (LW) across four evaluative criteria. Panels show: (**a**) naturalness, (**b**) aesthetics, (**c**) perceived hazard, and (**d**) need for improvement.

Figure 4 presents the mean perceptual scores for each scene (A–J), comparing images with LW and without LW across the four evaluated attributes. Table 3 summarizes the descriptive statistics (mean, median, and standard deviation) for each attribute and condition, providing an overall comparison of central tendency and response variability used in the statistical analyses.

**Table 3:** Summary statistics (mean, median, and standard deviation) of perceptual evaluations for images with and without LW, considering the four criteria assessed

Attribute	Group	Mean	Median (exp)	Standard Deviation (exp)
Aesthetics	With LW	2.20	2	0.71
	Without LW	2.47	3	0.77
Naturalness	With LW	2.53	3	0.65
	Without LW	2.41	3	0.83
Need for improvement	With LW	1.76	2	0.73
	Without LW	1.53	1	0.78
Hazard	With LW	1.78	2	0.70
	Without LW	1.71	2	0.75

### Correlations Among Perceptual Evaluation Attributes

To explore potential associations between the evaluated perceptual dimensions, Spearman’s rank correlation coefficients (ρ) were calculated for all pairwise combinations of Aesthetics, Naturalness, Hazard, and Need for Improvement (Table 4).

**Table 4:** Spearman correlation coefficients between the four perception attributes assessed in the study

Attribute	Aesthetics	Naturalness	Need for improvement	Hazard
**Aesthetics**	1.00	0.53	−0.29	−0.04
**Naturalness**	0.53	1.00	−0.37	0.04
**Need for improvement**	−0.29	−0.37	1.00	0.29
**Hazard**	−0.04	0.04	0.29	1.00

A moderate positive association was observed between Aesthetics and Naturalness (ρ = 0.53), indicating that scenes perceived as more aesthetically pleasing also tended to be judged as more natural. Conversely, Need for Improvement showed moderate negative correlations with both Aesthetics (ρ = −0.29) and Naturalness (ρ = −0.37), suggesting that images rated as less attractive or less natural were more frequently interpreted as requiring intervention.

Hazard exhibited weak associations with Aesthetics and Naturalness (ρ ≈ −0.04), indicating that perceived risk was largely independent of scenic quality or perceived naturalness. However, Hazard showed a positive correlation with Need for Improvement (ρ = 0.29), suggesting that environments interpreted as potentially risky were also more likely to be judged as requiring remediation.

Overall, the correlation structure indicates two complementary perceptual tendencies: one linking Aesthetic appeal and Naturalness, and another connecting risk perception with judgements of intervention. These correlations describe how perceptual attributes co-vary within respondents, whereas group comparisons assess differences in average ratings across respondent categories.

### Justifications for Perceptions of Hazard and Need for Improvement

**Fig. 5:**
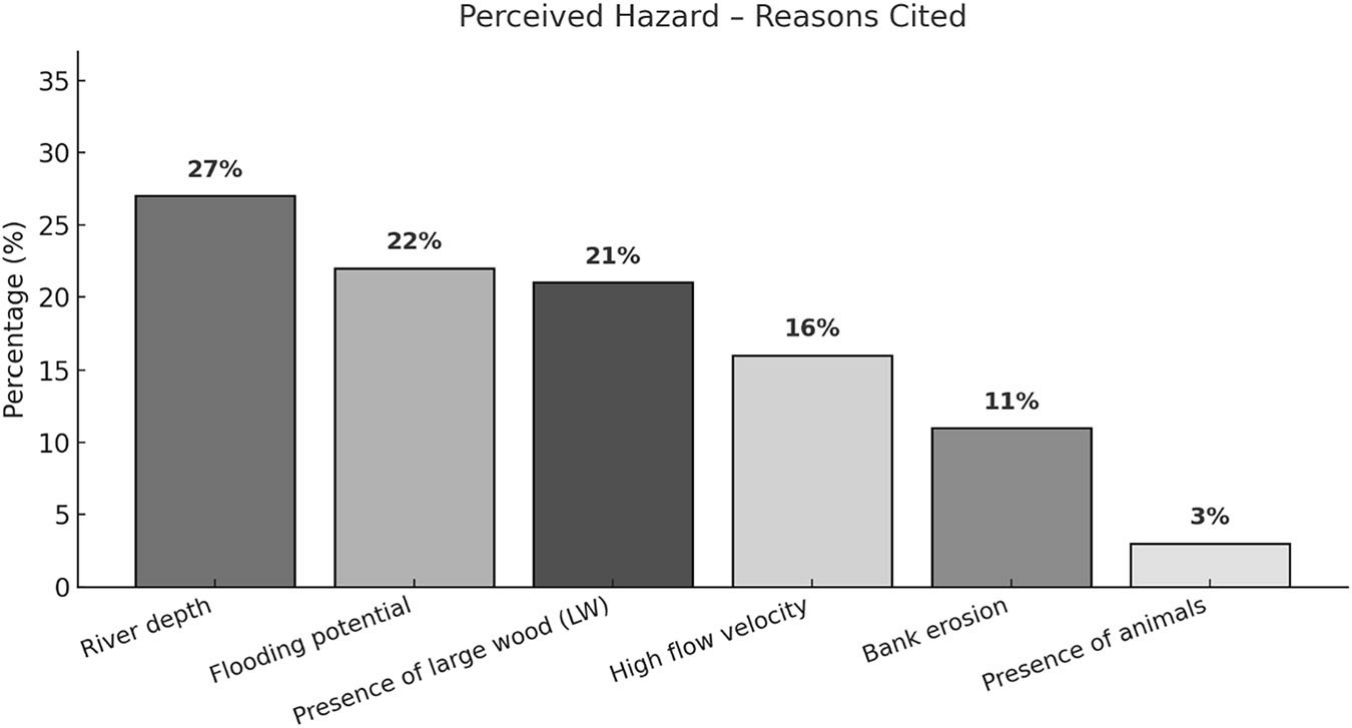
Main justification categories provided by participants who rated images as hazardous in the perception survey

**Fig. 6:**
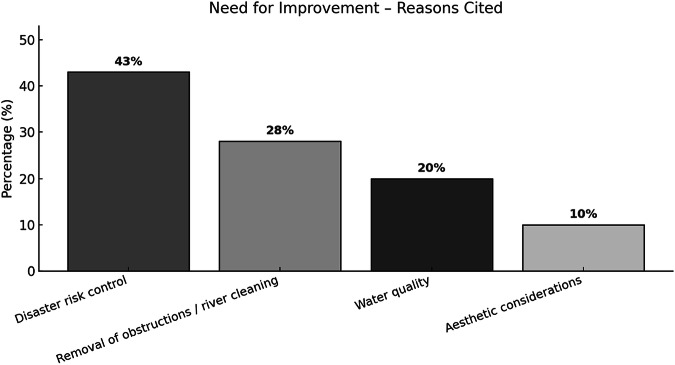
Main justification categories provided by participants who rated images as in Need of Improvement in the perception survey

As part of the questionnaire, respondents who rated any image with a score of 2 or 3 for the criteria “Hazard” or “Need for Improvement” were prompted to provide a justification for their assessment. The analysis of these open-ended responses revealed consistent patterns in the motivations expressed by participants (Figs. 5 and 6).

Regarding the perception of hazard (Fig. 5), the most frequently cited concern was river depth (27%), often associated with limited visibility of the riverbed or an inability to judge the safety for human use (e.g., bathing or crossing). Flooding potential followed closely (22%), particularly in cases where respondents associated morphology or riparian vegetation with possible overflow during extreme rainfall events. The *presence of LW* was also highlighted by 21% of respondents, commonly described as obstructive or potentially dangerous during high-flow conditions. Other reasons included *high flow velocity* (16%), *bank erosion* (11%), and, to a lesser extent, the *presence of animals* (3%), particularly venomous or predatory species.

In contrast, justifications related to the Need for Improvement revealed a strong emphasis on anthropic interventions (Fig. 6). The most recurrent motivation was the desire for disaster risk control (43%), encompassing flood prevention and mitigation measures. This is followed by requests for the removal of obstructions and river cleaning (28%). Water quality concerns accounted for 20% of the responses, frequently linked to perceived stagnation or pollution. Lastly, aesthetic considerations were mentioned by 10% of respondents, particularly in landscapes perceived as degraded, channelized, or lacking riparian vegetation.

These findings emphasize that the perceived risks and necessary interventions are not always based on ecological functionality but are frequently shaped by human safety, infrastructure concerns, and visual assessments. Notably, the presence of LW, a key component of fluvial ecosystems, emerged as a central factor in respondents’ perceptions of Hazard or Need for Improvement.

## Discussion

### Negative Perceptions and the Legacy of Hydraulic Paradigms

A substantial proportion of respondents in this study associated the presence of LW with hazard, disorder, and the need for intervention, indicating a predominantly risk-oriented interpretation of instream structural complexity. In our sample, 62.7% of participants rated LW-containing scenes as dangerous (scores 2 or 3), and 58.4% considered them to require improvement. Reported justifications frequently referred to flood risk, obstruction removal, and channel “cleaning,” with many participants explicitly linking wood accumulations to increased danger or reduced hydraulic efficiency. Notably, these tendencies were observed even among respondents with environmental training and public-sector experience, suggesting that disciplinary background alone does not fully mitigate generalized concerns regarding LW in river channels.

Such perceptions are consistent with patterns documented internationally. Cross-cultural photo-questionnaire studies conducted by Piégay et al. (2005) and Chin et al. (2008) demonstrated that river scenes containing wood were commonly judged as less aesthetic, more hazardous, and in greater need of intervention than wood-free channels, even among students and environmental professionals. More recent representative surveys indicate that this ambivalence persists at the population level, with LW frequently perceived simultaneously as natural and problematic, and safety considerations often outweighing recognition of ecological benefits (Gapinski et al., 2021). Similarly, studies conducted in regions recently affected by flooding show that direct or indirect exposure to flood impacts can amplify risk-based interpretations of instream features, reinforcing negative associations with LW accumulations (Dalu et al., 2022; Ruiz-Villanueva et al., 2018). Together, these findings suggest that the Brazilian patterns observed here reflect a broader and recurrent international tendency rather than a context-specific anomaly.

Beyond immediate risk perception, these responses also reflect a longer historical legacy in which rivers have been managed primarily as hydraulic infrastructure rather than ecological systems. Throughout the twentieth century, channelization, dredging, and systematic removal of instream wood were widely implemented to maximize conveyance efficiency and reduce perceived flood hazards. Such practices progressively normalized simplified, obstruction-free channels as the expected or “proper” state of rivers. This engineering-oriented framing, often described as a hydraulic or command-and-control paradigm, has been shown to influence both institutional decisions and public expectations, reinforcing the idea that structural complexity represents dysfunction rather than ecological function (Kondolf, 2006; Gurnell et al., 2012, 2018; Wohl, 2015, Wohl et al., 2019). From this perspective, negative evaluations of LW may be understood not only as immediate safety concerns but also as the outcome of historically embedded management narratives that equate order, clearance, and conveyance with river health.

These tendencies can be further interpreted through established theories of environmental risk perception and landscape aesthetics. As proposed by Slovic (1987), lay evaluations are often guided by intuitive and affective heuristics, in which visually salient cues of potential danger weigh more heavily than less visible or indirect ecological benefits. In visually based assessments such as photo-questionnaires, these mechanisms are likely reinforced, encouraging respondents to prioritize signs of obstruction, accumulation, or instability. This interpretation aligns with Nassauer’s (1995) concept of “messy ecosystems, orderly frames,” which explains why structurally complex but ecologically functional environments are frequently undervalued when they conflict with culturally embedded expectations of order, control, and maintenance. In riverine contexts, LW represents a paradigmatic example of such a feature, whose ecological benefits may be overshadowed by perceptions of visual disorder.

Overall, the convergence between our results and international evidence indicates the persistence of a perception gap between ecological function and social interpretation. Although fluvial science recognizes LW as a key driver of habitat formation, sediment retention, and hydrological regulation, public and professional evaluations often continue to privilege visually simplified and hydraulically efficient channels. This gap highlights the enduring influence of hydraulic paradigms in shaping how rivers are perceived and valued and provides the foundation for the more detailed multi-scale analysis presented in the following section.

### Multi-scale Patterns of Perception: Condition-level, Image-level, and Expertise Effects

Although the results discussed above indicate a generalized tendency to associate large wood (LW) with hazard and intervention demands, a more nuanced interpretation emerges when responses are examined across analytical scales. Rather than reflecting a uniformly negative judgement toward LW, our findings reveal a structured, multi-level pattern in which the magnitude and direction of perceptions vary depending on whether evaluations are aggregated by condition, by individual image, or by respondent background.

At the condition level, comparisons between scenes with and without LW showed statistically significant but relatively small differences. When ratings were averaged within respondents and contrasted using paired non-parametric tests, LW presence was associated with slightly higher naturalness scores but lower aesthetic evaluations and greater perceived need for intervention. Despite statistical significance, these effects were modest in magnitude, with overlapping distributions and comparable dispersion across conditions. This pattern indicates that the presence of wood alone does not determine perception in a deterministic manner, but exerts only a moderate influence within a broader set of visual and contextual cues. Similar condition-level contrasts have been reported in representative surveys and post-flood assessments, where LW tends to receive somewhat lower aesthetic and safety ratings without eliciting categorical rejection (Dalu et al., 2022; Wohl, 2016; Gapinski et al., 2021). Together, these findings suggest that average perceptual penalties associated with wood are generally incremental rather than extreme.

Greater variability emerged at the image level. Individual photographs elicited markedly divergent responses irrespective of LW presence, with some wood-free scenes receiving overwhelmingly positive evaluations and others being strongly rejected, while wood-containing scenes ranged from highly appreciated to negatively perceived. This heterogeneity indicates that contextual landscape attributes, such as riparian continuity, channel modification, visible infrastructure, and overall visual order, often outweigh the isolated effect of wood. Respondents, therefore, appear to evaluate riverscapes holistically rather than focusing on a single structural element. Previous photo-questionnaire studies have demonstrated the sensitivity of environmental judgements to framing, spatial scale, and surrounding land use, showing that in-channel wood may be interpreted either as natural complexity or as disorder depending on context (Le Lay et al., 2008, 2012; Ruiz-Villanueva et al., 2018). The pronounced contrasts observed among individual photographs in the present study are consistent with this stimulus-dependent behaviour.

Clearer differences were detected across disciplinary backgrounds and educational levels. Respondents trained in Environmental and Natural Resource Sciences consistently attributed higher naturalness and aesthetic values to LW scenes and reported lower hazard and intervention demands, whereas participants from other fields tended to express more cautious or negative evaluations. These patterns indicate that ecological literacy and professional familiarity with fluvial processes may mitigate predominantly risk-based interpretations of wood. Comparable effects of academic education on perceptions of instream wood have been documented previously, with more specialized training associated with greater acceptance of wood as a natural and functional component of rivers (Wyżga et al., 2009). This convergence supports the interpretation that perception is shaped not only by visual cues but also by the conceptual frameworks through which those cues are interpreted.

Taken together, these multi-scale patterns demonstrate that LW perception cannot be reduced to a simple binary opposition between presence and absence. Instead, evaluations emerge from the interaction between stimulus characteristics (image composition and landscape context), individual experience (e.g., exposure to flooding), and disciplinary knowledge. Recognizing this layered structure helps explain why statistically significant condition-level differences coexist with strong variability among images and why expertise exerts a stronger influence than employment sector or formal education alone. More broadly, these findings are consistent with the existence of a perception gap, in which the ecological functions of large wood are well established scientifically but only partially recognized socially (Wohl, 2015).

### Normalization of Absence and the Silenced Function of LW in Rivers

The multi-scale patterns described above indicate that negative evaluations of LW cannot be explained solely by immediate perceptions of hazard or visual disorder. Instead, they appear consistent with longer historical and experiential processes that have progressively shaped what is considered a “normal” or desirable river condition. In our sample, wood-free scenes generally received higher aesthetic and naturalness scores, suggesting that visually simplified and unobstructed channels are often implicitly treated as reference states. Similar tendencies have been documented in representative surveys and post-flood assessments, where respondents frequently associate cleared channels with safety and proper management (Gapinski et al., 2021; Dalu et al., 2022).

These perceptual baselines may reflect decades of river management practices emphasizing channel clearance, dredging, and systematic removal of instream structures. As discussed by Wohl et al. (2019), widespread extraction of LW during the twentieth century substantially reduced structural complexity in many rivers worldwide, promoting simplified morphologies that became socially familiar and visually normative. Over time, such transformations are consistent with a shifting baseline effect, in which historically altered environmental states become progressively internalized as natural conditions (Soga & Gaston, 2018). Within this framework, the absence of LW may be perceived as normal or desirable, whereas its presence is interpreted as anomalous or problematic.

Empirical perception studies provide further support for this interpretation. Chin et al. (2008) and Le Lay et al. (2008) demonstrated that respondents unfamiliar with natural river morphology often evaluate structurally complex channels less favourably, interpreting wood accumulations as signs of neglect rather than ecological functionality. Likewise, recent or recurrent flood experiences have been shown to amplify risk-oriented judgements of LW, reinforcing preferences for channel “cleaning” or removal (Ruiz-Villanueva et al., 2018; Dalu et al., 2022). The regional concentration of respondents in southern Brazil, where flood events are frequent, may therefore contribute to similar associations in the present study.

From a geomorphological and ecological perspective, however, these interpretations contrast with the documented functional role of LW. Instream wood enhances hydraulic retention, increases habitat heterogeneity, promotes sediment and organic matter storage, and supports channel–floodplain connectivity (Wohl, 2014; Beckman & Wohl, 2014; Gurnell et al., 2012, 2018). Consequently, features that may appear visually “messy” often correspond to ecologically functional states. The divergence between these ecological processes and their social interpretation has been described as a perception gap, in which scientifically recognized benefits are not fully reflected in public or professional judgements (Wohl, 2015).

Recent restoration initiatives demonstrate that alternative management approaches are feasible. Case studies from North America and Europe show that working with, rather than removing, LW can enhance geomorphic and ecological resilience while maintaining acceptable risk levels when appropriately designed and communicated (Ockelford et al., 2024). Such examples indicate that negative perceptions are not fixed but may evolve alongside changes in management practices and exposure to functioning, wood-rich riverscapes.

Taken together, these lines of evidence suggest that the perception gap observed in this study reflects the interaction between historically simplified baselines, disturbance memories, and limited familiarity with the ecological functions of LW. Recognizing these influences is essential for interpreting public responses and for designing communication and restoration strategies that better align visual expectations with ecological reality.

### Institutional Perception and the Role of Environmental Governance in Brazil

Although this study did not directly evaluate institutional procedures or regulatory decisions, the perceptual patterns observed among respondents suggest potential implications for environmental governance. Risk-centred interpretations of LW persisted even among participants with environmental training and professional experience, indicating that hazard-oriented framings may extend beyond lay audiences. Because many environmental management decisions rely partly on expert judgement and qualitative assessments, such perceptual tendencies may indirectly influence how riverscape elements are interpreted in planning and licensing contexts.

This consideration is particularly relevant given the composition of our sample. A substantial proportion of respondents were affiliated with the public sector or research institutions, groups directly involved in environmental licensing, monitoring, and river management activities. The presence of similar perceptual patterns within these professional profiles suggests that the limited recognition of LW functions is not confined to the general public but may also occur among actors who contribute to technical assessments. Consequently, the results indicate that the perception gap identified here has practical relevance for institutional contexts.

Similar perception–ecology mismatches have been documented internationally. Reviews of river management practice show that historically dominant hydraulic paradigms have often prioritized channel conveyance, flood control, and visual order, sometimes leading to systematic removal of instream structures and simplification of channel morphology (Gurnell et al., 2012; Wohl et al., 2018). In these settings, wood has frequently been framed primarily as debris or obstruction rather than as a functional geomorphic component. Comparative analyses of management strategies further demonstrate that working with, rather than removing, LW can enhance habitat complexity, sediment retention, and hydraulic heterogeneity, supporting more resilient river systems (Ockelford et al., 2024).

From a process-based perspective, these management trade-offs are not trivial. LW alters flow resistance, promotes energy dissipation, and affects water and sediment connectivity at multiple spatial scales. (Poeppl et al., 2023; Abatti et al., 2024). When such functions are not explicitly recognized in professional evaluation, the ecological consequences of removal may be underestimated. Within the Brazilian context, the present findings therefore suggest that strengthening the interface between scientific knowledge and applied management is essential. Increasing familiarity with the ecological and geomorphic roles of LW and incorporating process-based criteria into environmental licensing and restoration planning may help align institutional practice with contemporary river science. In this sense, improving ecological literacy among technical professionals becomes not only an educational objective but also a governance strategy for supporting more multifunctional and resilient riverscapes.

### Methodological Considerations and Limitations

Some limitations related to the sampling strategy should be acknowledged. Participation relied on voluntary recruitment through academic and professional networks, resulting in uneven regional representation and a higher proportion of respondents from southern Brazil. These characteristics may influence the generalizability of results to other socio-environmental contexts.

Visual framing and spatial scale of photographic stimuli also represent potential sources of bias. Perceptual judgements are sensitive to composition and context (Le Lay et al., 2012), and some LW images required closer framing to highlight wood accumulations. Consequently, observed contrasts may partly reflect contextual differences rather than wood presence alone. At the same time, these differences partly reflect intrinsic geomorphological and ecological characteristics of wood-rich environments rather than solely photographic choices. Large wood is more frequently retained in smaller, forested, or headwater channels with dense riparian vegetation, where shading, structural complexity, and reduced open-water visibility are characteristic features (Piégay & Gurnell, 1997; ; Wohl et al., 2019). Such environments often appear visually enclosed or less orderly, which may influence aesthetic and hazard-related judgements despite their ecological functionality (Wohl, 2015). Consequently, some perceptual contrasts observed between images with and without wood may reflect both natural environmental context and framing effects, and results should be interpreted with this combined influence in mind.

The use of a three-point Likert-type scale constitutes an additional limitation. Scales with relatively few response categories can retain acceptable reliability for attitudinal measurement, with only marginal gains from additional options (Matell & Jacoby, 1971), but reduced resolution may limit sensitivity to subtle contrasts. Continuous or visual analogue formats may capture finer distinctions (Le Lay et al., 2012). Thus, results should be interpreted as relative tendencies rather than precise quantitative magnitudes.

Finally, photo-based assessments rely on static visual cues and cannot reproduce the multisensory and experiential aspects of real-world environments. The cross-sectional design captures stated perceptions rather than behavioural or institutional decisions. Consequently, the findings indicate associations rather than causal relationships and should be interpreted within these methodological boundaries.

## Conclusions

This study examined respondents with different professional backgrounds' perceptions of LW in Brazilian rivers using a photo-questionnaire approach and identified a consistent gap between scientifically recognized ecological functions and socially expressed evaluations. Although LW plays an established role in promoting habitat complexity, hydraulic heterogeneity, and sediment dynamics, many respondents associated its presence primarily with hazard, disorder, or the need for intervention. These tendencies were observed not only among lay participants but also among individuals affiliated with environmental and public-sector professions.

Perceptual differences were statistically significant but generally modest at the condition level, while stronger variability emerged across individual images and respondent backgrounds. This multi-scale pattern indicates that LW perception is not determined solely by wood presence, but rather by the interaction between landscape context, visual cues, prior experiences, and disciplinary knowledge. In particular, respondents with training in Environmental and Natural Resource Sciences tended to attribute higher naturalness and aesthetic values to LW scenes and reported lower intervention demands, suggesting that ecological familiarity may moderate risk-oriented interpretations.

Taken together, these findings suggest that negative evaluations of LW reflect historically shaped expectations of simplified and visually orderly channels, which may not fully align with contemporary process-based river science. Improving communication of the ecological and geomorphic roles of LW, and increasing exposure to functioning wood-rich riverscapes, may therefore help reduce misunderstandings and support more informed assessments of river conditions.

## Data Availability

The data supporting the findings of this study are available from the corresponding author upon reasonable request.

## References

[CR1] Abatti BH, Michel GP, PoeppI RE, Fagundes MR, Paul LR, Zanandrea F (2024) The influence of large wood on sediment routing and flow characteristics: A study in a low-order stream in the southern Brazilian plateau. Geomorphology 459: 109398. 10.1016/j.geomorph.2024.109398

[CR2] Albagli S, Iwama AY (2022) Citizen science and the right to research: Building local knowledge of climate change impacts. Hum Soc Sci Commun 9(1):39. 10.1057/s41599-022-01040-8

[CR3] Ardoin NM, Bowers AW, Gaillard E (2020) Environmental education outcomes for conservation: A systematic review. Biol Conserv 241: 108224. 10.1016/j.biocon.2019.108224

[CR4] Arheimer B, Cudennec C, Castellarin A, Grimaldi S, Heal KV, Lupton C, Sarkar A, Tian F, Kileshye Onema J-M, Archfield S, Blöschl G, Borges Chaffe PL, Croke BFW, Dembélé M, Leong C, Mijic A, Mosquera GM, Nlend B, Olusola AO, Polo, MJ, … & et al. (2024) The IAHS Science for Solutions decade, with Hydrology Engaging Local People IN a Global world (HELPING). Hydrol Sci J. 10.1080/02626667.2024.2355202

[CR5] Arthington AH, Naiman RJ, McClain ME, Nilsson C (2010) Preserving the biodiversity and ecological services of rivers: New challenges and research opportunities. Freshw Biol 55(1):1–16. 10.1111/j.1365-2427.2009.02340.x

[CR6] Aryal K, Laudari HK, Neupane PR, Maraseni T (2021) Who shapes the environmental policy in the global south? Unpacking the reality of Nepal. Environ Sci Policy 121:78–88. 10.1016/j.envsci.2021.04.008

[CR7] Barros N, Cole JJ, Tranvik LJ, Prairie YT, Bastviken D, Huszar VLM, del Giorgio P, Roland F (2011) Carbon emission from hydroelectric reservoirs linked to reservoir age and latitude. Nat Geosci 4:593–596. 10.1038/ngeo1211

[CR8] Beckman ND, Wohl E (2014) Carbon storage in mountainous headwater streams: The role of old-growth forest and logjams. Water Resour Res 50:2376–2393. 10.1002/2013WR014167

[CR9] Bennett NJ (2016) Using perceptions as evidence to improve conservation and environmental management. Conserv Biol 30(3):582–592. 10.1111/cobi.12681 26801337 10.1111/cobi.12681

[CR10] Brierley G, Fryirs K, Cullum C, Tadaki M, Huang HQ, Blue B (2013) Reading the landscape: Integrating the theory and practice of geomorphology to develop place-based understandings of river systems. Prog Phys Geogr 37(5):601–621. 10.1177/0309133313490007

[CR11] Buytaert W, Zulkafli Z, Grainger S, Acosta L, Alemie TC, Bastiaensen J, De Bièvre B, Bhusal J, Clark J, Dewulf A, Foggin M, Hannah DM, Hergarten C, Isaeva A, Karpouzoglou T, Pandeya B, Paudel D, Sharma K, Steenhuis T, Zhumanova M (2014) Citizen science in hydrology and water resources: Opportunities for knowledge generation, ecosystem service management, and sustainable development. Front Earth Sci 2:26. 10.3389/feart.2014.00026

[CR12] Campagnolo K, Kobiyama M, Fan FM (2020) Panorama geral sobre estudos da influência dos detritos lenhosos na dinâmica de rios do mundo e do Brasil. Ciência e Natura, 42(Ed. Comemorativa – 40 anos). 10.5902/2179460X39228

[CR13] Campagnolo K, Kobiyama M, Fagundes MR, De Menezes D, Iroumé A, Michel GP, Rodrigues MF (2024) Influence of large wood dynamics on flow and channel morphology in a forest stream. Geomorphology 459: 109268. 10.1016/j.geomorph.2024.109268

[CR14] Chin A, Daniels MD, Urban MA, Piégay H, Gregory KJ, Bigler W, Butt AZ, Grable JL, Gregory SV, Lafrenz M, Laurencio LR, Wohl E (2008) Perceptions of wood in rivers and challenges for stream restoration in the United States. Environ Manag 41(6):893–903. 10.1007/s00267-008-9075-9 10.1007/s00267-008-9075-9 18305987

[CR15] Comiti F, Lucía A, Rickenmann D (2016) Large wood recruitment and transport during large floods: A review. Geomorphology 269:23–39. 10.1016/j.geomorph.2016.06.016

[CR62] Dalu MTB, Cuthbert RN, Ragimana P, Gunter AW, Dondofema F, Dalu T (2022) Assessing human perceptions towards large wood in river ecosystems following flooding experiences. River Research and Applications 38(7):1296–1304. 10.1002/rra.4009

[CR16] Fearnside PM (1999) Social impacts of Brazil’s Tucuruí Dam: A review. Environ Manag 24(4):483–495. 10.1007/s002679900248 10.1007/s002679900248 10501861

[CR17] Fernández-Llamazares Á, Díaz-Reviriego I, Guèze M, Cabeza M, Pyhälä A, Reyes-García V (2016) Local perceptions as a guide for the sustainable management of natural resources: Empirical evidence from a small-scale society in Bolivian Amazonia. Ecol Soc, 21(1). 10.5751/ES-08092-210102 10.5751/ES-08092-210102 PMC5029546 27660639

[CR18] Fearnside PM, Pueyo S (2012) Greenhouse-gas emissions from tropical dams. Nat Clim Change 2:382–384

[CR19] Fritz S, See L, Carlson T et al. (2019) Citizen science and the United Nations Sustainable Development Goals. Nat Sustain 2:922–930. 10.1038/s41893-019-0390-3

[CR20] Galia T, Poledniková Z, Škarpich V (2024) Impact of large wood on sediment (dis)connectivity in a meandering river. Geomorphology 453: 109153. 10.1016/j.geomorph.2024.109153.

[CR21] Gapinski CM, Hermes J, von Haaren C (2021) Why people like or dislike large wood in rivers—A representative survey of the general public in Germany. River Res Appl. 10.1002/rra.3743

[CR22] Gippel CJ (1995) Environmental hydraulics of large woody debris in streams and rivers. J Environ Eng 121(5):388–395. 10.1061/(ASCE)0733-9372(1995)121:5(388).

[CR23] Gregory R, Fischhoff B, McDaniels T (2005) Acceptable input: Using decision analysis to guide public policy deliberations. Decis Anal 2(1):4–16. 10.1287/deca.1050.0035.

[CR24] Gurnell AM, Bertoldi W, Corenblit D (2012) Changing river channels: The roles of hydrological processes, plants and pioneer fluvial landforms in humid temperate, mixed load, gravel bed rivers. Earth-Sci Rev 111(1-2):129–141. 10.1016/j.earscirev.2011.11.005.

[CR25] Gurnell A, England J, Burgess-Gamble L (2018) Trees and wood: Working with natural river processes. Water Environ J 33(3):342–352. 10.1111/wej.12426.

[CR26] Gurnell AM, Bertoldi W (2020) Wood in fluvial systems. In Treatise on Geomorphology (2nd ed., pp. 1–33). Elsevier Inc. 10.1016/B978-0-12-409548-9.12415-7

[CR27] Hester ET, Doyle MW (2008) In-stream geomorphic structures as drivers of hyporheic exchange. Water Resour Res 44(3):W03417. 10.1029/2006WR005810.

[CR28] Holmes J, Clark R (2008) Enhancing the use of science in environmental policy-making and regulation. Environ Sci Policy 11(8):702–711. 10.1016/j.envsci.2008.08.004.

[CR29] Hooke J (2023) Morphodynamics of active meandering rivers reviewed in a hierarchy of spatial and temporal scales. Geomorphology 439: 108825. 10.1016/j.geomorph.2023.108825

[CR30] Kondolf GM (2006) River restoration and meanders. Ecol Soc, 11(2), Article 42. 10.5751/ES-01795-110242

[CR31] Le Lay Y-F, Piégay H, Gregory KJ, Chin A, Dolédec S (2008) Variations in cross-cultural perception of riverscapes in relation to in-channel wood. Trans Inst Br Geog 33(2):268–287. 10.1111/j.1475-5661.2008.00297.x

[CR32] Le Lay Y-F, Cottet M, Piégay H, Rivière-Honegger A (2012) Ground imagery and environmental perception: Using photo-questionnaires to evaluate river management strategies. In P. E. Carbonneau & H Piégay (Eds.), Fluvial Remote Sensing for Science and Management. Wiley. 10.1002/9781119940791.ch18

[CR33] Lyn DA, Cooper TJ, Condon CA, Gan L (2007) Factors in debris accumulation at bridge piers (FHWA/IN/JTRP-2006/36). Purdue University, Joint Transportation Research Program. 10.5703/1288284313364

[CR34] Matell MS, Jacoby J (1971) Is there an optimal number of alternatives for Likert-scale items? Study I: Reliability and validity. Educ Psychol Meas 31(3):657–674. 10.1177/001316447103100307

[CR35] McKinley DC, Miller-Rushing AJ, Ballard HL, Bonney R, Brown H, Cook-Patton SC, Evans DM, French RA, Parrish JK, Phillips TB, Ryan SF, Shanley LA, Shirk JL, Stepenuck KF, Weltzin JF, Wiggins A, Boyle OD, Briggs RD, Chapin SF, Hewitt DA, Soukup MA (2017) Citizen science can improve conservation science, natural resource management, and environmental protection. Biol Conserv 208:15–28. 10.1016/j.biocon.2016.05.015

[CR36] Montanari A, Young G, Savenije HHG, Hughes D, Wagener T, Ren LL, Koutsoyiannis D, Cudennec C, Grimaldi S, Blöschl G, Sivapalan M, Beven K, Gupta H, Arheimer B, Huang Y, Schumann A, Post D, Srinivasan V, Boegh E, Hipsey M (2013) “Panta Rhei—Everything Flows”: Change in hydrology and society—The IAHS Scientific Decade 2013–2022. Hydrol Sci J 58(6):1256–1275. 10.1080/02626667.2013.809088

[CR37] Ockelford A et al. (2024) Working with wood in rivers in the Western United States. River Res Appl. 10.1002/rra.4331

[CR38] Pahl-Wostl C (2007) Transitions towards adaptive management of water facing climate and global change. Water Resour Manag 21(1):49–62. 10.1007/s11269-006-9040-4

[CR39] Piégay H, Gurnell AM (1997) Large woody debris and river geomorphological pattern: Examples from S.E. France and S. England. Geomorphology 19:99–116. 10.1016/S0169-555X(96)00045-1

[CR40] Piégay H, Gregory KJ, Bondarev V, Chin A, Dahlstrom N, Elosegi A, Gregory SV, Joshi V, Mutz M, Rinaldi M, Wyzga B, Zawiejska J (2005) Public perception as a barrier to introducing wood in rivers for restoration purposes. Environ Manag 36(5):665–674. 10.1007/s00267-004-0092-z 10.1007/s00267-004-0092-z 16215648

[CR41] Pinto CF, Agra JUM, Furley TH (2017) Uso da madeira de eucalipto na recuperação de rios: Projeto ReNaturalize. O Pap 78(8):106–113

[CR42] Poeppl RE, Perez JE, Fergg H, Morche D (2023) Introducing indices to assess the effects of in-stream large wood on water and sediment connectivity in small streams. Geomorphology 439: 108936. 10.1016/j.geomorph.2023.108936

[CR43] Poeppl RE, Fryirs KA, Tunnicliffe J, Brierley GJ (2020) Managing sediment (dis)connectivity in fluvial systems. Sci Total Environ 736: 139627. 10.1016/j.scitotenv.2020.139627 32485383 10.1016/j.scitotenv.2020.139627

[CR44] Reed MS (2008) Stakeholder participation for environmental management: A literature review. Biol Conserv 141(10):2417–2431. 10.1016/j.biocon.2008.07.014

[CR45] Ruiz-Villanueva V, Díez-Herrero A, Bladé E, Bodoque JM (2014) Large wood transport as significant influence on flood risk in a mountain village. Nat Hazards 74:967–987. 10.1007/s11069-014-1222-4

[CR46] Ruiz-Villanueva V et al. (2017) Large wood clogging during floods in a gravel-bed river: The Długopole bridge in the Czarny Dunajec River, Poland. Earth Surf Process Landforms. 10.1002/esp.4091

[CR47] Ruiz-Villanueva V, Díez-Herrero A, García JA, Ollero A, Piégay H, Stoffel M (2018) Does the public’s negative perception towards wood in rivers relate to recent impact of flooding experiencing?. Sci Total Environ 635:294–307. 10.1016/j.scitotenv.2018.04.096 29674258 10.1016/j.scitotenv.2018.04.096

[CR48] Starkey E, Parkin G, Birkinshaw S, Large A, Quinn P, Gibson C (2017) Demonstrating the value of community-based (‘citizen science’) observations for catchment modelling and characterisation. J Hydrol 548:801–817. 10.1016/j.jhydrol.2017.03.019

[CR49] Slovic P (1987) Perception of risk. Science 236(4799):280–285. 10.1126/science.3563507 3563507 10.1126/science.3563507

[CR50] Soga M, Gaston KJ (2018) Shifting baseline syndrome: Causes, consequences, and implications. Front Ecol Environ 16(4):222–230. 10.1002/fee.1794

[CR51] Swanson FJ, Gregory SV, Iroumé A, Ruiz-Villanueva V, Wohl E (2020) Reflections on the history of research on large wood in rivers. Earth Surf Process Landf 45(1):4–22. 10.1002/esp.4814

[CR52] United Nations Development Programme (UNDP) (2023) Environmental citizen science and its effects on participants, governance, and innovation: Evidence of two small-scale experiments. UNDP

[CR53] Vanelli FM, Kobiyama M (2021) How can socio-hydrology contribute to natural disaster risk reduction?. Hydrol Sci J 66(7):1047–1057. 10.1080/02626667.2021.1967356

[CR54] Vannote RL, Minshall GW, Cummins KW, Sedell JR, Cushing CE (1980) The River Continuum Concept. Can J Fish Aquat Sci 37(1):130–137. 10.1139/f80-017

[CR55] Verdonschot PFM, Verdonschot RCM (2024) Ecological functions and management of large wood in fluvial systems. Curr For Rep 10(1):39–55. 10.1007/s40725-023-00209-x

[CR56] Wohl E (2015) Of wood and rivers: Bridging the perception gap. Wiley Interdiscip Rev: Water 2(5):445–459. 10.1002/wat2.1076

[CR57] Wohl E (2016) Spatial heterogeneity as a component of river geomorphic complexity. Prog Phys Geogr 40(4):598–615. 10.1177/0309133316658615

[CR58] Wohl E, Beckman ND (2014) Leaky rivers: Implications of the loss of longitudinal fluvial disconnectivity in headwater streams. Geomorphology 205:27–35. 10.1016/j.geomorph.2011.10.022

[CR59] Wohl E, Kramer N, Ruiz-Villanueva V, Scott DN, Comiti F, Gurnell AM, Piégay H, Liniger KB, Jaeger KL, Walters DM, Fausch KD (2019) The natural wood regime in rivers. BioScience 69(4):259–273. 10.1093/biosci/biz013

[CR60] Wyżga B, Zawiejska J, Le Lay Y-F (2009) Influence of academic education on the perception of wood in watercourses. J Environ Manag 90(1):587–603. 10.1016/j.jenvman.2007.12.013 10.1016/j.jenvman.2007.12.013 18262329

[CR61] Zischg AP et al. (2018) Modelling spatiotemporal dynamics of large wood recruitment, transport, and deposition at the river reach scale during extreme floods. Water 10(9):1134. 10.3390/w10091134

